# ASSESSMENT OF OLDER ADULTS’ BEHAVIORAL SYMPTOMS AND THEIR SOCIAL BEHAVIORS USING SURVEYS AND A TECHNOLOGY PLATFORM

**DOI:** 10.1093/geroni/igad104.1837

**Published:** 2023-12-21

**Authors:** Wan-Tai Au-Yeung, Lyndsey Miller, Allison Lindauer, Nathaniel Rodrigues, Cierra Leon Guerrero, Sarah Gothard, Zach Beattie, Jeffrey Kaye

**Affiliations:** Oregon Health & Science University, Portland, Oregon, United States; Oregon Health & Science University, Portland, Oregon, United States; Oregon Health & Science University, Portland, Oregon, United States; Oregon Health & Science University, Portland, Oregon, United States; Oregon Health & Science University, Portland, Oregon, United States; Oregon Health & Science University, Portland, Oregon, United States; Oregon Health & Science University, Portland, Oregon, United States; Oregon Health & Science University, Portland, Oregon, United States

## Abstract

Behavioral symptoms are highly prevalent during the course of cognitive decline associated with dementia. Studies have shown that the social environment may affect these symptoms. Data sources examining the effect of environment come from two ongoing studies: 1) MOnitoring DEmentia-Related Agitation Using Technology Evaluations (MODERATE), focusing on agitation in individuals with later-stage dementia, and 2) Monitoring Apathy, Depression and Anxiety Using Technology Evaluations (ADA), focusing on apathy, depression and anxiety in older adults with or without cognitive decline. To date, a total of 9 dyads have enrolled in MODERATE while a total of 9 participants have enrolled in ADA. Caregivers (MODERATE) and participants with cognitive impairment (ADA) report symptoms via weekly online surveys. MODERATE integrates a modified Cohen-Mansfield Agitation Inventory-Short Form series of questions regarding weekly agitation-related levels/events. The ADA weekly online survey consists of Withdrawal-Apathy-Vigor, a subscale from Geriatric Depression Scale-30, a 4-item depression subscale from Patient-Reported Outcomes Measurement Information System-29 (PROMIS-29), and a 4-item anxiety subscale from PROMIS-29. A multi-sensor array (bed pressure mats, motion sensors, and wearables) is deployed in homes to objectively measure participants’ behaviors. With this array, duration of time-out of home, duration of simultaneous activities detected in more than one room, and the total number of occupants and pets within the home are extracted. Additionally, weekly online surveys inquire as to whether participants have been out of town or have overnight visitors. Associations between social activity and behavioral symptoms are obtained combining digital behavioral-activity biomarkers with frequently reported online behavioral data.

